# Development and application of a targeted MISCP-S intervention model for psychiatric hospitals

**DOI:** 10.3389/fcimb.2026.1799863

**Published:** 2026-10-09

**Authors:** Jing Du, Xin Jiang, Qiwen Li, Xin Liu, Gang Qing, Yan Yang, Mi Yang

**Affiliations:** The Fourth People’s Hospital of Chengdu, Chengdu, Sichuan, China

**Keywords:** appropriateness of antimicrobial use, healthcare-associated infection (HAI), MISCP-S intervention model, multidrug-resistant organisms (MDROs), pathogen detection specimen submission rate for HAI diagnosis, psychiatric specialty hospital

## Abstract

**Objective:**

A multi-modal intervention model (MISCP-S) was constructed based on the characteristics of psychiatric medical institutions, to evaluate its impact on the pathogen detection specimen submission rate related to healthcare-associated infection diagnosis and the effect of rational antibacterial use.

**Methods:**

In a large tertiary psychiatric hospital, we established the MISCP-S Intervention Model (Multidisciplinary Collaboration, Informatization and Intelligence, Stratified Training, Constraints and Incentives, and Process Optimization) tailored to specialty-specific infection control challenges. 1343 HAI cases were collected as the pre-intervention group from January 2022 to November 2023, and 677 cases were collected as the post-intervention group from March 2024 to July 2025.

**Results:**

The pathogen detection specimen submission rate increased significantly from 69.47% to 98.38% (P < 0.05), with the appropriateness rate of tests ordered reaching 100%. This improvement was most pronounced in departments with previously low submission rates, such as the acute psychiatric department (P < 0.05). Multivariate logistic regression identified the intervention, patient location in ICU/internal medicine/chronic care units, and urinary tract infection as significant facilitating factors for specimen submission (all P < 0.05). The therapeutic antimicrobial use rate decreased from 16.03% to 14.61% (P < 0.05). The overall detection rate of multidrug-resistant organisms (MDROs) decreased from 37.44% to 33.74% (P < 0.05). Notably, detection rates of methicillin-resistant *Staphylococcus aureus* (MRSA) and carbapenem-resistant Pseudomonas aeruginosa (CRPA) decreased significantly (both P < 0.05). In contrast, the rate of carbapenem-resistant Enterobacterales (CRE) increased from 14.35% to 23.56% (P < 0.05). The proportions of blood cultures and bronchoscopy samples rose from 14.94% to 22.34% and from 3.88% to 8.63%, respectively (both P < 0.05).

**Conclusion:**

The MISCP-S intervention model appears to be associated with improvements in specimen submission quality, reductions in overall MDRO detection rates, and enhanced antimicrobial stewardship in psychiatric settings. These findings suggest that the model may offer a replicable framework for similar healthcare institutions.

## 1 Introduction

Antimicrobial overuse has driven the emergence and spread of bacterial resistance, which has become a major global public health challenge. In 2019, approximately five million deaths were attributable to AMR-related infections worldwide (GBD 2019 Antimicrobial Resistance Collaborators, 2022; Li et al., 2023; World Health Organization, 2025). Promoting rational antimicrobial use and curbing the transmission of resistance have therefore become critical priorities both domestically and internationally. Pathogen detection specimen submission is a key component in precisely guiding antimicrobial therapy and interrupting the spread of resistant organisms, and its clinical implementation quality directly influences infection prevention and control outcomes. In October 2021, the National Institute of Hospital Administration of the National Health Commission issued the “Guiding Opinion on the Special Action to Increase the Pathogen Detection Specimen Submission Rate Prior to Antimicrobial Therapy in Hospitalized Patients” (Document No. [2021]198), which for the first time explicitly set a target that the pathogen detection specimen submission rate related to HAI diagnosis should be no less than 90% (National Health Commission of the People's Republic of China, 2021).

Psychiatric inpatients are at high risk of healthcare-associated infections (HAIs) due to prolonged hospital stays, elevated iatrogenic infection risks, and varying degrees of cognitive and behavioral impairments (Zhou et al., 2020). However, this population presents with challenges including insidious infection symptoms and poor cooperation with specimen collection. Meanwhile, psychiatric healthcare professionals exhibit relative deficiencies in infection recognition, appropriate specimen selection and standardized sampling, and rational antimicrobial use, compounded by inadequate interdepartmental collaboration. These unique challenges have resulted in psychiatric institutions achieving submission rates far below the national target. Therefore, this study took the improvement of the pathogen detection specimen submission rate for HAI diagnosis as its entry point and implemented the MISCP-S intervention model. The aim was to systematically optimize submission workflows and clinical practices, enhance the submission rate, and further explore the synergistic effects of this intervention on rational antimicrobial use and the prevention and control of antimicrobial resistance, thereby providing practical evidence and reference for psychiatric hospitals, geriatric hospitals, nursing homes, and other similar healthcare institutions.

## 2 Materials and methods

### 2.1 Samples

HAI cases from January 2022 to July 2025 at a tertiary Grade A psychiatric hospital were screened using the “Hospital Infection and Infectious Disease Real-Time Surveillance Platform” (Chengdu Xiaochouyu Network Information Technology Co., Ltd.).

### 2.2 Inclusion criteria

HAI cases meeting the diagnostic criteria of the *healthcare-associated infection Diagnostic Standards (Trial)* issued by the Ministry of Health in 2001 (Ministry of Health of the People's Republic of China, 2003), with complete clinical information including infection site, and clinical manifestations, were identified and confirmed jointly by two full-time infection control professionals from the Hospital Infection Management Department. The confirmation of HAI diagnosis was performed blinded to whether the case belonged to the pre-intervention or post-intervention period.

### 2.3 Exclusion criteria

HAI cases with incomplete clinical information (e.g., unclear infection site or missing).

### 2.4 Classification methods

This study was a retrospective study with a pre-post comparative design. Based on the timing of intervention implementation, 1343 HAI cases were collected as a pre-intervention group from January 2022 to November 2023, and 677 cases were collected as a post-intervention group from March 2024 to July 2025. The period from December 2023 to February 2024 was defined as an intervention-adaptation phase and was excluded from the statistical analysis. This was primarily because the construction of the intervention model, training of healthcare staff, and full implementation of the intervention measures required a transition period; failure to establish an adaptation phase could result in incomplete implementation and, consequently, compromise the accuracy of the evaluation of the intervention effect.

### 2.5 Construction and implementation of the MISCP-S intervention model

Through survey-based assessment and systematic identification of core infection prevention and control challenges specific to psychiatric settings, a MISCP-S intervention model was constructed with precise alignment between the identified challenges and the intervention modules. Target improvement benchmarks were established, including pathogen detection specimen submission rates for HAI of ≥90% and 100% accuracy for submitted specimen items. Led by the Department of Infection Management, a multidisciplinary working group comprising the medical, nursing, laboratory, pharmacy, logistics, and information technology departments was formed to develop a targeted action plan, clarify departmental responsibilities (as detailed in **Table 1**), and establish a dedicated enterprise WeChat communication group to ensure real-time issue resolution.

**Table 1:** Modules of the MISCP-S intervention model.

Module	Department(s)	Responsibilities
Multidisciplinary Collaboration	Infection Management Medical Affairs, Nursing Laboratory Pharmacy Information Technology Logistics Support	Collaborative implementation based on action plan and departmental responsibilities.
Informatization and Intelligence	Information Technology	Enhanced HIS with automated high-risk patient flagging and symptom-based specimen collection guidance.
Stratified Training	Infection Management	Hospital-wide training delivered in a tiered, stratified manner for all personnel.
Constraint and Incentive	Infection Management	Routine monitoring and monthly performance review and reporting.
Process Optimization	Infection Management Medical Affairs Nursing Laboratory Pharmacy Information Technology Logistics Support	Optimized processes across specimen collection, submission, and laboratory reporting.

Building on the hospital’s existing information infrastructure, the Laboratory Information System (LIS) dictionary was updated and standardized to ensure data accuracy. Furthermore, automated pop-up reminders were embedded into both the Electronic Medical Record (EMR) system and the Hospital Infection and Infectious Disease Real-Time Surveillance Platform. These reminders were triggered when physicians recorded infection indicators or prescribed antimicrobial agents. The surveillance platform automatically generated monthly reports on the quality of microbiological specimen submission for HAI, enabling full-process monitoring and management.

### 2.6 Outcome measures

Primary outcomes: Pathogen detection specimen submission rate related to HAI diagnosis (number of cases with specimen submission/total number of HAI cases × 100%), and the appropriateness rate of tests ordered (number of cases with appropriately ordered tests/number of cases with specimen submission × 100%).Appropriateness was determined jointly by two full-time infection control professionals based on the Diagnostic Criteria for Healthcare-Associated Infections (Trial) (Ministry of Health of the People's Republic of China, 2003) and the Guidelines for Collection and Transport of Clinical Microbiology Specimens WS/T 640-2018 (National Health Commission of the People's Republic of China, 2018). A test was considered inappropriate if the submitted specimen type did not match the suspected site of infection (e.g., urine culture submitted for a lower respiratory tract infection). Repeated isolates of the same pathogen from the same infection site within 14 days were counted once.

Secondary outcomes: defined as the number of MDRO isolates divided by the total number of pathogens isolated during the same period × 100%. This metric represents the proportion of identified isolates that were MDROs (a measure of detection proportion), rather than the incidence or prevalence of MDRO infection or acquisition. Therefore, changes in this measure should be interpreted as reflecting shifts in the composition of detected pathogens, not necessarily changes in MDRO transmission dynamics.

### 2.7 Statistical analysis

Statistical analyses were performed using SPSS (version 22.0). Categorical variables were expressed as counts (percentages), and comparisons of proportions were conducted using the chi-square test, corrected chi-square test, or Fisher’s exact test, as appropriate.

In the multivariable logistic regression analysis, the dependent variable was whether a specimen was submitted for pathogen detection related to the HAI diagnosis. Covariates were predetermined based on clinical plausibility and previous literature, including age, number of underlying diseases, infection site, clinical department, and intervention period. All prespecified variables were simultaneously entered into the multivariable model. Multicollinearity among independent variables was assessed using the variance inflation factor (VIF), with a VIF > 10 considered indicative of severe collinearity.

This study is reported in accordance with the STROBE (STrengthening the Reporting of OBservational studies in Epidemiology) guidelines.

## 3 Results

### 3.1 General characteristics

A total of 2,020 cases of healthcare-associated infections (HAIs) were included in this study, with 1,343 cases in the pre-intervention group and 677 cases in the post-intervention group. There were no statistically significant differences between the two groups in terms of sex, age, or departmental distribution. However, significant differences were observed in the number of underlying diseases and sites of infection (P < 0.05) (**Table 2**).

**Table 2:** Comparison of baseline characteristics between the pre-intervention and post-intervention groups.

Category	Pre-intervention	Post-intervention	*χ²*	*P*
gentle			2.473	0.116
man	738	347		
woman	605	330		
Age group (years)			2.692	0.442
0-17	44	15		
18-44	79	45		
45-64	380	182		
≥65	840	435		
Number of underlying diseases			10.31	0.018
0	797	365		
1	436	237		
2	85	50		
≥3	25	25		
Department			6.707	0.349
Acute Psychiatry	287	141		
ICU	23	20		
Internal Medicine	318	146		
Geriatrics	497	246		
Addiction Medicine	107	63		
Chronic Psychiatry	73	45		
Clinical Psychology	38	16		
Site of infection			11.347	0.01
Lower respiratory tract infection	941	521		
Urinary tract infection	202	81		
Upper respiratory tract infection	75	32		
Other infection sites	125	43		

### 3.2 Logistic regression analysis of pathogen detection specimen submission rate related to HAI diagnosis

To clarify the effects and relative contributions of influencing factors on the pathogen detection specimen submission rate for HAI diagnosis before and after the intervention, univariate analyses were first performed. Variables that remained statistically significant were subsequently included in a regression model. A binary multivariate logistic regression analysis was conducted with pathogen detection specimen submission rate related to HAI diagnosis as the dependent variable (0 = no submission, 1 = submission). Independent variables included patient age group, number of underlying diseases, site of infection, clinical department, and intervention (pre- vs. post-intervention).

The results showed that patient age and the number of underlying diseases were not associated with specimen submission. The post-intervention time-period was identified as a factor associated with higher odds of pathogen-detection specimen submission related to HAI diagnosis (OR = 33.446, 95% CI: 18.1–61.8). Using the acute psychiatric department as the reference group (which had the lowest pre-intervention submission rate), the ICU (OR = 12.627, 95% CI: 1.613–98.828), the department of Internal Medicine (OR = 4.694, 95% CI: 2.809–7.846) and the chronic psychiatry department (OR = 2.253, 95% CI: 1.248–4.067) were identified as facilitating factors. With lower respiratory tract infection as the reference category (the predominant infection type hospital-wide), urinary tract infection was also a facilitating factor for pathogen detection specimen submission (OR = 11.363, 95% CI: 6.022–21.439). These results are presented in **Table 3**. A linear regression model was used to assess multicollinearity between the two independent variables, clinical department and site of infection. The variance inflation factor (VIF) values were below 10, (tolerance: >0.1), indicating no severe multicollinearity between these two variables; therefore, no adjustment was required.

**Table 3:** Multivariable logistic regression analysis of factors associated with pathogen detection specimen submission rate related to HAI diagnosis.

Independent variable	*β*	Wald χ²	*P*	OR (95% CI)
Intervention period (post vs. pre)	3.510	125.54	<0.001	33.446 (18.100–61.800)
Department (reference: Acute Psychiatry)		75.913	<0.001	
ICU	2.536	5.835	0.016	12.627 (1.613–98.828)
Internal Medicine	1.546	34.814	<0.001	4.694 (2.809–7.846)
Geriatrics	0.035	0.026	0.873	1.036 (0.672–1.597)
Addiction Medicine	−0. 197	0.724	0.395	0.821 (0.522–1.293)
Chronic Psychiatry	0.812	7.261	0.007	2.253 (1.248–4.067)
Clinical Psychology	−0.825	3.821	0.051	0.438 (0.191–1.002)
Age group (years; reference: 0–17)		3.356	0.340	
18–44	−0.630	2.101	0.147	0.533 (0.227–1.248)
45–64	−0.217	0.282	0.596	0.805 (0.361–1.794)
≥65	−0.269	0.376	0.540	0.764 (0.324–1.803)
Site of infection (reference: Lower respiratory tract infection)		56.796	<0.001	
Urinary tract infection	2.430	56.297	<0.001	11.363 (6.022–21.439)
Upper respiratory tract infection	0.108	0.162	0.688	1.114 (0.659–1.882)
Other infection sites	0.264	1.441	0.230	1.302 (0.846–2.004)
Number of underlying diseases (reference: 0)		2.085	0.555	
1	−0. 135	0.850	0.357	0.874 (0.656–1.164)
2	−0. 100	0.118	0.732	0.905 (0.510–1.606)
≥ 3	0.654	0.994	0.319	1.922 (0.532–6.947)
Constant	0.553	1.874	0.171	1.738

### 3.3 Pathogen detection specimen submission rate related to HAI diagnosis before and after the intervention

During the post-intervention period, the hospital-wide pathogen-detection-specimen submission rate for HAI diagnosis was associated with a significant increase from 69.47% to 98.38%, and the appropriateness rate of submitted specimens increased from 86.63% to 100% (both P < 0.05). The most pronounced improvements in specimen submission rates were observed in the acute psychiatric department (45.30% to 95.74%), the addiction medicine department (54.21% to 100%), and the clinical psychology department (50.00% to 93.75%) (all P < 0.05), as shown in **Table 4**. After the intervention, infection control professionals reviewed the submitted specimen information. When inappropriate specimen submissions were identified, the attending physicians were reminded to resubmit. As a result, no inappropriate submissions were recorded during the post-intervention period.

**Table 4:** Comparison of Pathogen detection specimen submission rate related to HAI diagnosis before and after Intervention.

	Pre-intervention (n=1343)					Post-intervention (n=677)						
Department	Appropriate (n)	Inappropriate^*^ (n)	Not submitted (n)	Submission rate (%)	Appropriateness rate (%)	Appropriate (n)	Inappropriate^*^ (n)	Not submitted (n)	Submission rate (%)	Appropriateness rate (%)	Statistic	*P*
Hospital-wide	933	144	266	69.47	86.63	666	0	11	98.38	100.00	227.928	<0.001
ICU	22	0	1	95.65	100.00	20	0	0	100.00	100.00	-*	0.535
Internal Medicine	278	19	21	87.42	93.60	146	0	0	100.00	100.00	20.097	<0.001
Geriatrics	332	69	96	66.80	82.79	242	0	5	98.37	100.00	94.041	<0.001
Acute Psychiatry	130	69	88	45.30	65.33	135	0	4	95.74	100.00	102.049	<0.001
Addiction Medicine	58	20	29	54.21	74.36	63	0	0	100.00	100.00	40.534	<0.001
Clinical Psychology	19	2	17	50.00	90.48	15	0	1	93.75	100.00	7.461	0.006
Chronic Diseases	54	9	10	73.97	85.71	45	0	0	100.00	100.00	13.960	<0.001

Inappropriate^*^: The submitted specimen was inconsistent with the infection site of patients with HAIs. For example, urine specimens submitted for lower respiratory tract infections were deemed inappropriate.

-*: Minimum expected count <1, Fisher’s exact test was used.

Specimen Submission Rate = (Number of cases with specimens appropriately submitted)/(The total number of HAI cases) × 100%; The pathogen detection specimen submission rate for HAI diagnosis was calculated using the number of HAI episodes as the denominator.

Appropriateness Rate = (Number of cases with specimens appropriately submitted)/(Number of cases with specimens appropriately submitted+ Number of inappropriate samples submitted for inspection) × 100%.

### 3.4 Therapeutic antimicrobial use rate among hospitalized patients before and after the intervention

The post-intervention observation period was associated with an overall reduction in the hospital-wide therapeutic-antimicrobial-use rate among inpatients from 16.03% to 14.61% (P < 0.05). Significant reductions were observed in the geriatrics department (20.76% to 17.23%) and the addiction medicine department (10.04% to 8.03%) (both P < 0.05). In contrast, the therapeutic antimicrobial use rate in the ICU increased from 53.11% to 60.66% (P < 0.05), as shown in **Table 5**.

**Table 5:** Comparison of therapeutic antimicrobial use rates among hospitalized patients by department before and after the intervention.

	Pre-intervention			Post-intervention				
Department	HP (n)	PCA (n)	PCA/HP (%)	HP (n)	PCA (n)	PCA/HP (%)	*χ* ^2^	*P*
Hospital-wide	28 573	4 580	16.03	22 673	3 313	14.61	19.481	<0.001
ICU	610	324	53.11	544	330	60.66	4.084	0.043
Internal Medicine (3 units)	4 867	2 012	41.34	3 648	1 567	42.96	2.330	0.135
Geriatrics (5 units)	6 815	1 415	20.76	5 049	870	17.23	23.265	<0.001
Acute Psychiatry (5 units)	7 150	504	7.05	5 409	340	6.29	2.861	0.091
Addiction Medicine (2 units)	2 788	280	10.04	2 266	182	8.03	6.088	0.014
Clinical Psychology (4 units)	5 927	75	1.27	5 170	56	1.08	0.786	0.375
Chronic Rehabilitation (2 units)	1 564	114	7.29	1 025	91	8.88	2.144	0.143

HP, Hospitalized patients; PCA, Patients receiving antimicrobials. Statistical comparisons were performed between pre- and post-intervention periods for each department.

### 3.5 Detection rate of MDROs before and after the intervention

After the intervention, the overall hospital-wide detection rate of MDROs decreased from 37.44% to 33.74% (P < 0.05). Specifically, MRSA detection rates declined significantly from 67.26% to 51.31%, and CRPA detection rates decreased from 80.00% to 51.32% (both P < 0.05). In contrast, the detection rate of CRE increased from 14.35% to 23.56% (P < 0.05), as shown in **Table 6**.

**Table 6:** Comparison of MDRO detection Rate among inpatients before and after the intervention.

	Pre-intervention			Post-intervention				
	MDRO isolates (n)	Total isolates (n)	Detection rate (%)	MDRO isolates (n)	Total isolates (n)	Detection rate (%)	Statistic	*P*
MRSA	304	452	67.26	137	267	51.31	17.996	<0.001
CRAB	317	897	35.34	137	422	32.46	1.051	0.35
CRPA	272	340	80.00	39	76	51.32	27.085	<0.001
CRE	162	1129	14.35	127	539	23.56	21.619	<0.001
Total	1055	2818	37.44	440	1304	33.74	5.267	0.022

Statistical comparisons were performed between pre- and post-intervention periods for each MDRO.

### 3.6 Proportion of infection-related pathogen specimen types before and after the intervention

After the intervention, the proportion of blood specimens increased from 14.94% to 22.34%, while the proportion of bronchoscopic specimens (including bronchoalveolar lavage fluid, bronchial lavage fluid, and fiberoptic bronchoscopy specimens) increased from 3.88% to 8.63%; both differences were statistically significant (P < 0.05), as shown in **Table 7**.

**Table 7:** Proportion of infection-related pathogen specimen types before and after the intervention.

	Pre-intervention		Post-intervention			
Specimen type	Specimens (n)	Proportion (%)	Specimens (n)	Proportion (%)	Statistic	*P*
Sputum	8910	50.89	5129	44.05	130.932	<0.001
Urine	2184	12.47	1095	9.40	114.697	<0.001
Blood	2616	14.94	2601	22.34	260.485	<0.001
Bronchoscopic specimens	679	3.88	1005	8.63	290.316	<0.001
Others	3121	17.82	1814	15.58	25.071	<0.001
Total	17510	100.00	11644	100.00	–	–

Statistical comparisons were performed between pre- and post-intervention periods for each specimen type.

## 4 Discussion

Since 2000 to 2015, global per capita antimicrobial consumption increased by 90.9% (Klein et al., 2021). Antimicrobial agents are essential for treating infectious diseases; however, inappropriate or excessive use may lead to treatment failure, prolonged hospitalization, increased healthcare costs, and waste of medical resources (Sun et al., 2021; Yan et al., 2021). Timely and standardized pathogen detection at the onset of infection is of great significance for the accurate diagnosis of healthcare-associated infections (HAIs) and for guiding rational antimicrobial therapy. Such practices can reduce unnecessary antibiotic use, representing a key strategy to curb the emergence and spread of bacterial resistance, and a core objective of antimicrobial stewardship (AMS) programs (Bassetti et al., 2022).

At present, interventions aimed at improving pathogen detection in psychiatric healthcare settings are understudied both domestically and internationally, and a mature, well-targeted, and comprehensively evaluated model is currently lacking. This study addresses key challenges in infection control within psychiatric specialty settings by developing and implementing the MISCP-S intervention model tailored to psychiatric healthcare institutions. This model can enhance the pathogen detection specimen submission rate and standardization related to hospital-acquired infection diagnosis, promote rational use of antimicrobial agents, and reduce the overall detection rate of multidrug-resistant organisms (MDROs). These findings suggest that process management is a critical component of effective HAI prevention and control (Ahmed et al., 2019).Furthermore, it narrowed inter-departmental quality disparities and achieved homogenization of medical quality, aligning well with the objectives of the special initiative and the core goals of hospital management.

### 4.1 Targeted innovation and practical value of the MISCP-S intervention mode

The core pain points of infection prevention and control in psychiatric medical institutions mainly include difficulties in the management of special patient populations, closed clinical settings, insufficient infection prevention and control capabilities among healthcare workers, and inadequate inter-departmental collaboration mechanisms. The MISCP-S intervention model precisely addresses these challenges through five major modules tailored to the characteristics of psychiatric specialties. First, multi-departmental collaboration breaks the dilemma of “the infection control department working in isolation”. Standardized workflows help improve the efficiency of collaboration among hospital departments (Rusjan and Kiauta, 2019). Additional support measures, such as cleaning and disinfection of closed wards and emergency response by security personnel, are implemented to ensure that resources are allocated toward specialty-specific priorities. Second, informatization support transforms “passive testing” into “active triggering”, compensating for psychiatrists’ tendency to prioritise psychiatric symptoms over infection signals. Third, stratified training addresses issues including a monotonous knowledge structure, a lack of interdisciplinary knowledge, and insufficient capabilities in infection diagnosis and treatment; following the intervention, the accuracy of ordered testing items reached 100%, effectively reversing issues of inappropriate sampling and specimen transportation. Fourth, the constraint-and-incentive mechanism shifts pressure from individuals to departments, maintaining pathogen submission rates consistently above 95%. Fifth, process optimization tailored to the characteristics of psychiatric patients—such as implementing a three-tier sampling pathway and the healthcare-associated infection (HAI) prevention and control protocol for specimen transportation and handover in closed wards—substantially enhances operational feasibility.

Overall, the MISCP-S intervention model establishes a closed-loop framework of “problem identification - model development - intervention implementation - evaluation and optimization” (**Figures 1**, **2**), effectively bridging interdepartmental disparities in healthcare quality and promoting standardized, homogeneous quality improvement across the hospital. These findings are generally consistent with those reported by Lijiang Zhang et al. (Xu et al., 2025; Zhang et al., 2026) in studies aimed at improving pathogen detection specimen submission, further supporting the notion that refined management models may be effective in enhancing pathogen detection specimen submission and antimicrobial stewardship.

**Figure 1:**
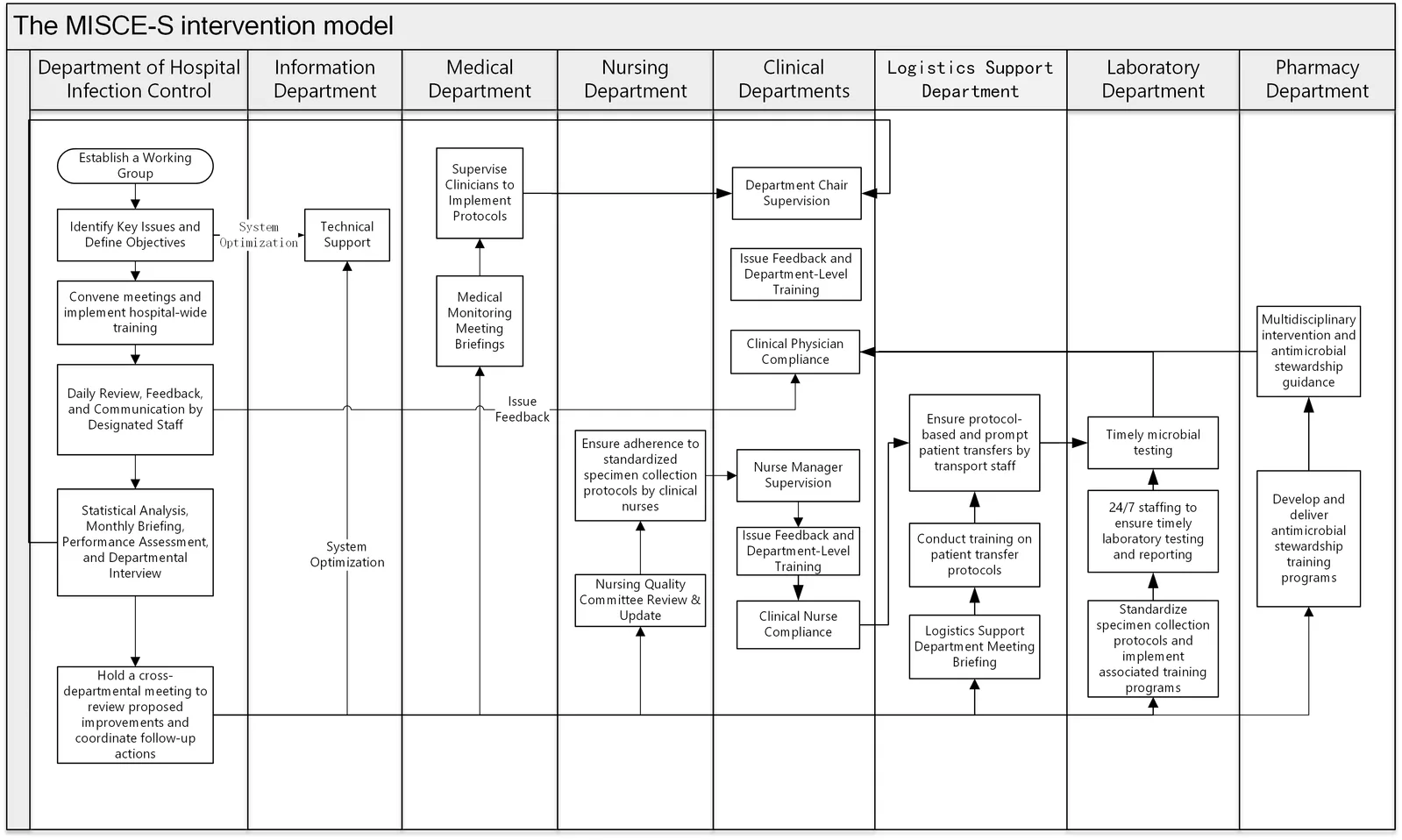
The MISCP-S intervention model.

**Figure 2:**
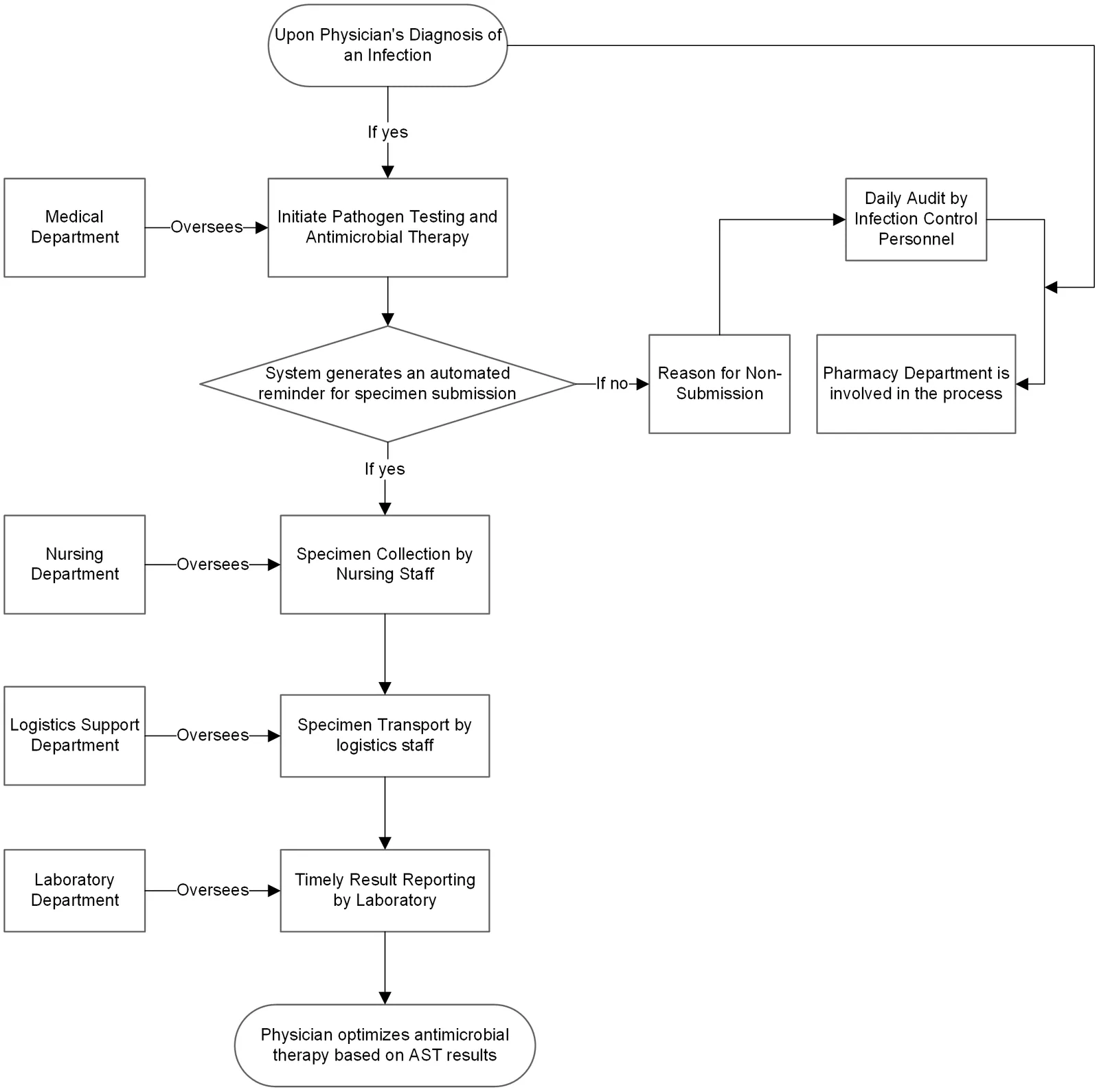
Flowchart of pathogen detection specimen submission for HAIs in psychiatric hospitals.

### 4.2 Synergistic effects of the intervention on antimicrobial use and AMR control

With the substantial increase in the pathogen detection specimen submission rate, the proportion of cases with accurate etiological identification and antimicrobial susceptibility results increased correspondingly. This enabled clinicians to select appropriate antimicrobial agents based on susceptibility testing, potentially reducing unnecessary empirical antimicrobial use and contributing to improved patient outcomes and prognosis (Liu et al., 2023), while also helping to reduce unnecessary antimicrobial consumption. In this study, the hospital-wide rate of therapeutic antimicrobial use among inpatients decreased by 1.42 percentage points post-intervention, with more pronounced reductions observed in the Geriatrics and Addiction Medicine Departments. This change may be associated with improved diagnostic accuracy. Before the intervention, the inability to identify pathogens frequently led to empiric antimicrobial therapy. Following the intervention, the increased specimen submission rate provided clinicians with definitive pathogen detection results, thereby helping to avoid unnecessary antibiotic use in cases of viral infections or non-infectious conditions. Furthermore, the reduction may be partially attributable to a “test-first” approach: for patients with mild symptoms, antimicrobial therapy was deferred pending test results. During this waiting period, a subset of patients remained untreated, some of whom ultimately did not require antibiotics due to negative findings. These observations suggest a possible association between precise pathogen detection and targeted antimicrobial therapy.

During the current monitoring period, the antimicrobial utilization rate in the ICU showed an upward trend. Based on a multidimensional analysis, the main contributing factors may include the following. First, the department’s admission volume continued to expand, with both the total number of patients and patient-days in the ICU increasing, thereby raising the baseline population receiving antimicrobial therapy. Second, the overall severity of admitted patients increased, with a growing number of critically ill, elderly, or immunocompromised patients transferred from internal medicine and geriatrics departments, who often presented with multiple underlying comorbidities. These patients had a high incidence of pulmonary, bloodstream, and urinary tract infections, leading to a marked increase in cases requiring anti-infective therapy. Third, the hospital has vigorously promoted the development of the critical care medicine department, with annual expansion of medical staffing, enhanced life-support equipment, and improved treatment technologies, leading to the widespread use of invasive procedures such as mechanical ventilation, vascular access, and continuous renal replacement therapy (CRRT). Fourth, the risk of colonization and cross-transmission of multidrug-resistant organisms (MDROs) increased, with a greater number of transferred patients carrying resistant strains, which prolonged the duration of anti-infective therapy and escalated the use of broad-spectrum or reserved antimicrobial agents.

Following the intervention, the overall hospital-wide MDRO detection rate decreased from 37.44% to 33.74% (P < 0.05). It is important to note that this metric reflects the proportion of MDROs among all detected pathogens (a detection proportion), rather than the true incidence or prevalence of MDRO infection. The substantial increase in specimen submission rate (from 69.47% to 98.38%) and the shift toward more sterile specimens (blood and bronchoscopic samples) may have increased the detection of previously unidentified colonizing or infecting organisms (Chen et al., 2024), which could theoretically inflate rather than reduce the MDRO detection proportion. Nevertheless, the concurrent decline in MRSA and CRPA detection proportions, alongside the overall reduction, suggests a potential positive association between improved diagnostic stewardship and reduced selection pressure. However, causal inferences regarding MDRO transmission reduction cannot be drawn from these data alone.”

### 4.3 Interpretation of the “paradox” of increased CRE detection and future intervention directions

Following the intervention, the CRE detection rate at our institution increased from 14.35% to 23.56% (P < 0.05), in contrast to the declining trends observed for overall MDRO, MRSA, and CRPA detection rates. It is important to emphasize that the following explanations for the increased CRE detection are speculative and are offered as hypotheses rather than demonstrated mechanisms. Several factors may account for this observation. First, long-term surveillance data from the CHINET network (Guo et al., 2025) indicate a fluctuating upward trend in carbapenem resistance among Enterobacterales in China, and our findings are broadly consistent with this national pattern. Second, the significant increase in the proportion of sterile specimens, including blood and bronchoscopy samples, along with enhanced laboratory diagnostic capacity, facilitated the accurate identification of previously occult CRE infections. Third, the expansion of ICU services—with the transfer of critically ill patients from geriatric psychiatry and internal medicine departments—may have concentrated antimicrobial selection pressure on high-risk patients. Concurrently, the increased frequency of invasive procedures in critically ill ICU patients may have further elevated the risk of CRE colonization and infection. Fourth, the inherent high-risk characteristics of patients in psychiatric specialty settings were compounded by prolonged hospital stays, multiple comorbidities, dependence on healthcare staff for daily care, and reduced compliance with infection control measures in clinically relevant situations. This is supported by domestic studies in neuropsychiatric hospitals, which suggest that CRE can readily spread within such settings (Yue et al., 2025).However, confirming these hypotheses and establishing a direct causal link will require dedicated future studies with stronger designs, including molecular epidemiology to track transmission pathways.

### 4.4 Limitations and future perspectives

This study was conducted at a single center; therefore, the sample’s representativeness is limited, which may constrain the generalizability and feasibility of the findings when applied to other settings. The disparate sample sizes of the pre-intervention (n=1,343) and post-intervention (n=677) groups may potentially affect the statistical power and robustness of the findings. This study has inherent design limitations, including the absence of random allocation and dynamic assessment. Additionally, the 3-month adaptation period may have led to an underestimation of the immediate effects of certain interventions that began to take effect earlier, and the potential influence of unmeasured contemporaneous confounding factors—such as differences in case composition between the two study periods—cannot be entirely ruled out. Furthermore, secondary outcomes, including the therapeutic antimicrobial use rate and the MDRO detection rate, were not subjected to stratified analyses due to incomplete data collection at the subgroup level; therefore, a direct causal link between the intervention and the observed reductions in these secondary outcomes could not be further established. Previous studies have demonstrated that informatization substantially enhances the efficiency of HAI management and serves as a powerful tool for promoting the rational clinical use of antimicrobial agents (Zhang et al., 2018; Ahmed et al., 2019; Ma et al., 2022; Xu et al., 2025). However, the level of informatization in psychiatric healthcare institutions remains relatively underdeveloped. At the present institution, the information system has not yet enabled automated comparison of specimen collection and antimicrobial administration times, making it difficult to accurately identify non-compliant practices, such as administering antimicrobials before pathogen testing. Furthermore, the pre-intervention phase of this study was influenced by the “Class B disease, Class B measures” policy implemented during the COVID-19 pandemic, which resulted in data fluctuations in December 2022, including a marked increase in HAI and a notable decline in pathogen submission rates. Nevertheless, the duration of this impact was short and is unlikely to have materially affected the overall results.

Future research will aim to enhance the external validity of the findings by conducting multicenter collaborative studies, adopting randomized controlled designs with dynamic evaluation of intervention effects during adaptation periods, and incorporating propensity score matching to control for contemporaneous confounding factors. These approaches will enable more precise validation of the impact of the MISCP-S intervention model and mitigate methodological limitations. Further efforts will focus on advancing informatization and intelligent systems to achieve cross-platform data integration and AI-assisted infection identification; developing multidisciplinary, closed-loop prevention and control models tailored to speciality characteristics; and strengthening regional collaboration and workforce training to promote management standardization. Collectively, these strategies are expected to continuously improve the precision and scientific rigor of HAI management in psychiatric hospitals.

## Data Availability

The original contributions presented in the study are included in the article/**Supplementary Material**. Further inquiries can be directed to the corresponding author.

## References

[B1] Ahmed E. Ahmad M. Othman S. (2019). Business process improvement methods in healthcare: a comparative study. Int. J. Health Care Qual. Assur. 32, 887–908. doi: 10.1108/IJHCQA-07-2017-0116 31195926

[B2] Bassetti S. Tschudin-Sutter S. Egli A. Osthoff M. (2022). Optimizing antibiotic therapies to reduce the risk of bacterial resistance. Eur. J. Intern. Med. 99, 7–12. doi: 10.1016/j.ejim.2022.01.029 35074246

[B3] Chen X. Li S. Xie X. Wu S. Zhang Y. Zhang W. . (2024). Concordance between specimens submitted for pathogen detection and target infection sites in the diagnosis of healthcare-associated infections. Chin. J. Nosocomiol 34, 455–459.

[B4] GBD 2019 Antimicrobial Resistance Collaborators (2022). Global mortality associated with 33 bacterial pathogens in 2019: a systematic analysis for the Global Burden of Disease Study 2019. Lancet 400, 2221–2248. doi: 10.1016/S0140-6736(22)02185-7 36423648 PMC9763654

[B5] Guo Y. Ding L. Hu F. Zhu D. Wang F. Tian Y. . (2025). CHINET antimicrobial resistance surveillance report in China, 2024. Chin. J. Infect. Chemother. 25, 597–607. doi: 10.16718/j.1009-7708.2025.06.002

[B6] Klein E. Y. Milkowska-Shibata M. Tseng K. K. Sharland M. Gandra S. Pulcini C. . (2021). Assessment of WHO antibiotic consumption and access targets in 76 countries, 2000–15: an analysis of pharmaceutical sales data. Lancet Infect. Dis. 21, 107–115. doi: 10.1016/S1473-3099(20)30332-7 32717205

[B7] Li Z. Zou R. Yu M. Liu W. Xu X. Wu F. . (2023). The role of quality control circles in improving the pathogen detection specimen submission rate prior to antimicrobial therapy in a hospital. Jiangsu Health System Manage. 34, 75–78.

[B8] Liu Y. Yang H. Shi J. Yu H. (2023). Effectiveness of comprehensive interventions in improving the pathogen detection specimen submission rate prior to antimicrobial therapy for hospitalized patients. Chin. J. Nosocomiol 33, 3024–3028.

[B9] Ma X. Chen X. Yao H. Liu Y. Xu X. Zhao S. . (2022). Current status of management and application of the indicator for pathogen detection specimen submission rate prior to antimicrobial therapy. Chin. J. Nosocomiol 32, 936–940.

[B10] Ministry of Health of the People's Republic of China (2003). Diagnostic criteria for healthcare-associated infections (Trial). Modern Pract. Med. 15, 460–465.

[B11] National Health Commission of the People's Republic of China (2018). WS/T 640-2018: Specimen collection and transport in clinical microbiology. 1–20.

[B12] National Health Commission of the People's Republic of China (2021). Guiding Opinions on the Special Action to Improve the Pathogen Detection Specimen Submission Rate Before AnVtimicrobial Therapy for Hospitalized Patients (National Health Medical Research Letter [2021] No. 198). Available online at: https://www.qiluhospital.com/uploadfile/2022/0120/20220120105510227.pdf (Accessed January 20, 2022).

[B13] Rusjan B. Kiauta M. (2019). Improving healthcare through process standardization: a general hospital case study. Int. J. Health Care Qual. Assur. 32, 459–469. doi: 10.1108/IJHCQA-06-2018-0142 31017068

[B14] Sun F. Wang L. Guo Q. Wang A. He Z. Xu M. . (2021). Study on the direct economic burden of patients with healthcare-associated infections based on DRG. Chin. J. Infect. Control 20, 725–729.

[B15] World Health Organization (2025). Global Antibiotic Resistance and Use Surveillance System (GLASS) Report 2025 (Geneva: World Health Organization). Available online at: https://appliedsciencecommons.net/artifacts/31978940/global-antibiotic-resistance-surveillance-report-2025/32878741/ (Accessed December 11, 2025).

[B16] Xu L. Chen H. Zhou Y. Liu D. (2025). A hospital infection management department-led intervention to improve the pathogen submission rate before antimicrobial therapy using a FOCUS-PDCA model. Infect. Drug Resist. 18, 5043–5055. doi: 10.2147/IDR.S547418 41000264 PMC12459385

[B17] Yan Y. Zhang X. Fu Z. Wang Y. Sun W. Liu C. . (2021). Analysis of pathogen detection and antimicrobial use in 40 medical institutions in Zhengzhou city. Chin. J. Disinfect 38, 189–191,194. doi: 10.11726/j.issn.1001-7658.2021.03.010

[B18] Yue L. Zhu X. Kuang Y. Lin Y. Liu H. (2025). Drug resistance and epidemiological analysis of carbapenem-resistant enterobacteriaceae in a brain and psychiatric hospital of south china. J. Clin. Lab. Anal. 39, e70073. doi: 10.1002/jcla.70073 40607763 PMC12329348

[B19] Zhang L. Xu Z. Wang Y. Meng Y. Ning K. Wang J. . (2026). Lean management enhances antimicrobial stewardship in a Chinese psychiatric hospital: a quality improvement study on aetiological submission rates. BMJ Open Qual. 15, e003981. doi: 10.1136/bmjoq-2025-003981 42082283 PMC13141241

[B20] Zhang L. Yao M. Li Y. (2018). Improving the pathogen detection specimen submission rate prior to antimicrobial use by applying PDCA cycle based on informatization. China Health Industry. 15, 170–172. doi: 10.16659/j.cnki.1672-5654.2018.29.170

[B21] Zhou W. Zhang W. Zeng W. Wang Y. Li Q. Jiang L. (2020). Risk and management strategies for healthcare-associated infections in psychiatric inpatients. Modern Hospitals 20, 1783–1785.

