# Supplementary material for: Development and application of a targeted MISCP-S intervention model for psychiatric hospitals
Source: Front Cell Infect Microbiol. 2026 Oct 9;16:1799863. doi: 10.3389/fcimb.2026.1799863 (PMC13690548; doi:10.3389/fcimb.2026.1799863)
Supplement: Supplementary file 1 [file Table1.docx]

**Table 1. Modules of the MISCP-S intervention model.**

| **Module** | **Department(s)** | **Responsibilities** |
| --- | --- | --- |
| **Multidisciplinary**  **Collaboration** | Infection Management | Overall coordination, technical guidance, supervision of corrective actions, and outcome tracking. |
|  | Medical Affairs, Nursing | Implementation of the action plan. |
|  | Laboratory | Optimization and refinement of testing and reporting workflows and related measures. |
|  | Pharmacy | Intervention for cases without specimen submission and provision of AMS guidance. |
|  | Information Technology | Informatics and technical support. |
|  | Logistics Support | Specimen transportation, cleaning and disinfection of closed wards, and emergency response by security personnel. |
| **Informatization and Intelligence** | Information Technology | The hospital's existing information system was upgraded to integrate specialized data fields, such as psychiatric symptom scores (agitation and cognitive impairment levels), sampling compliance grading, ward closure attributes and psychotropic medication administration records. It automatically flagged high-risk patients for specimen collection and provided tailored sampling schemes based on their psychiatric symptoms. |
| **Stratified Training** | Infection Management | **Hospital-wide training:** Mandatory training (online/onsite) had the following departmental thresholds: ≥80% participation and 100% accuracy. Results were linked to performance evaluations.  **Department-level training**: Department heads organised cascaded training, adapting hospital-level content to department-specific scenarios (e.g., specimen collection strategies for acutely uncooperative psychiatric patients) to provide targeted instruction.  **Role-specific training**: Targeted training programs were delivered to physicians, nurses, laboratory technicians and logistics staff respectively. Physician training focused on early identification of comorbid somatic infections in psychiatric patients, psychotropic-antibacterial drug interactions and specialty-specific anti-infective indications. Nurse training covered communication skills for vulnerable populations, emergency response to sampling-related adverse events and infection control in closed wards. Laboratory technicians received training on laboratory quality control and test reporting protocols, while logistics staff were trained on specimen transportation protocols, routine ward disinfection and patient daily hygiene management. |
| **Constraint and Incentive** | Infection Management | **Routine monitoring:** Designated personnel performed daily reviews of HAI case report cards and specimen submission records. Joint interventions with clinical pharmacists were conducted for non-submission cases. Specialized case discussions were held for failed sampling and irrational antibacterial use, and departmental implementation was supervised.  **Performance assessment and feedback:** Key benchmarks (e.g., HAI-related specimen submission/ compliance rates/the incidence of sampling-related healthcare worker occupational exposure) were reported monthly; underperformance triggered management reviews, with outcomes impacting departmental performance and director accountability. |
| **Process Optimization** | Infection Management  Medical Affairs  Nursing  Laboratory  Pharmacy  Information Technology  Logistics Support | **Sampling stage:** To standardise practices, we developed a Psychiatric Patient Microbiological Specimen Collection Guideline that introduces a three-step workflow (communication, assessment, and alternative sampling—e.g., blood when sputum collection is infeasible) for uncooperative patients.It specified targeted sampling protocols for patients with cognitive impairment, sedated patients and those at high agitation risk. Additionally, to improve nighttime infection detection, we optimized clinical-infection management linkages and mandated an infection screening protocol for psychiatrists.  **Submission/transport stage:** The HAI prevention and control protocol for specimen transportation and handover in closed wards was optimized. Specialized priority-transport containers were provided, and collected specimens were directly handed over to transport staff by medical personnel for real-time delivery.  **Feedback stage:** We introduced laboratory night shifts to ensure two verification rounds by 11:00 and 21:00 on weekdays. Positive results triggered immediate, simultaneous notification to attending physicians via phone and system alerts. |
