# Supplementary material for: Development and application of a targeted MISCP-S intervention model for psychiatric hospitals
Source: Front Cell Infect Microbiol. 2026 Oct 9;16:1799863. doi: 10.3389/fcimb.2026.1799863 (PMC13690548; doi:10.3389/fcimb.2026.1799863)
Supplement: Supplementary file 2 [file Table2.docx]

Monthly P‑chart for rates of incorrect specimen submissions and non‑submissions, January 2022 – July 2025

Rate of unsubmitted and incorrectly submitted samples（%）

| Month | Rate of unsubmitted and incorrectly submitted samples（%） | The center line (CL) | Upper control limits (UCL) | lower control limits (LCL) |
| --- | --- | --- | --- | --- |
| 2022-01 | 21.6216 | 20.8113 | 34.9688 | 6.65379 |
| 2022-02 | 33.9286 | 20.8113 | 37.0858 | 4.53676 |
| 2022-03 | 31.6667 | 20.8113 | 36.534 | 5.0886 |
| 2022-04 | 21.875 | 20.8113 | 36.0347 | 5.58786 |
| 2022-05 | 26.4151 | 20.8113 | 37.5401 | 4.0825 |
| 2022-06 | 23.8806 | 20.8113 | 35.69 | 5.93259 |
| 2022-07 | 12.5 | 20.8113 | 36.0347 | 5.58786 |
| 2022-08 | 12.2449 | 20.8113 | 38.2095 | 3.41309 |
| 2022-09 | 26.3158 | 20.8113 | 34.7813 | 6.84131 |
| 2022-10 | 19.3548 | 20.8113 | 36.2783 | 5.34427 |
| 2022-11 | 23.8095 | 20.8113 | 39.6035 | 2.01908 |
| 2022-12 | 60.2649 | 20.8113 | 30.7222 | 10.9004 |
| 2023-01 | 32.4324 | 20.8113 | 40.833 | 0.789562 |
| 2023-02 | 38.4615 | 20.8113 | 40.3129 | 1.3097 |
| 2023-03 | 30.3571 | 20.8113 | 37.0858 | 4.53676 |
| 2023-04 | 34.5455 | 20.8113 | 37.2331 | 4.38948 |
| 2023-05 | 29.5082 | 20.8113 | 36.4046 | 5.21801 |
| 2023-06 | 37.931 | 20.8113 | 43.4266 | 0 |
| 2023-07 | 21.0526 | 20.8113 | 40.5678 | 1.05476 |
| 2023-08 | 27.7778 | 20.8113 | 41.1092 | 0.513387 |
| 2023-09 | 25 | 20.8113 | 37.7002 | 3.92241 |
| 2023-10 | 41.6667 | 20.8113 | 36.534 | 5.0886 |
| 2023-11 | 27.4194 | 20.8113 | 36.2783 | 5.34427 |
| 2023-12 | 18.4783 | 20.8113 | 33.5085 | 8.11407 |
| 2024-01 | 20.8791 | 20.8113 | 33.5781 | 8.0445 |
| 2024-02 | 23.0769 | 20.8113 | 35.9172 | 5.70542 |
| 2024-03 | 4.44444 | 20.8113 | 38.9663 | 2.65629 |
| 2024-04 | 5 | 20.8113 | 40.0676 | 1.55501 |
| 2024-05 | 0 | 20.8113 | 40.5678 | 1.05476 |
| 2024-06 | 2.5 | 20.8113 | 40.0676 | 1.55501 |
| 2024-07 | 0 | 20.8113 | 41.3971 | 0.225459 |
| 2024-08 | 2.77778 | 20.8113 | 41.1092 | 0.513387 |
| 2024-09 | 0 | 20.8113 | 43.4266 | 0 |
| 2024-10 | 0 | 20.8113 | 42.685 | 0 |
| 2024-11 | 4.44444 | 20.8113 | 38.9663 | 2.65629 |
| 2024-12 | 0 | 20.8113 | 39.8313 | 1.79129 |
| 2025-01 | 0 | 20.8113 | 37.0858 | 4.53676 |
| 2025-02 | 1.96078 | 20.8113 | 37.8649 | 3.75764 |
| 2025-03 | 2.27273 | 20.8113 | 39.1714 | 2.45115 |
| 2025-04 | 2.5641 | 20.8113 | 40.3129 | 1.3097 |
| 2025-05 | 0 | 20.8113 | 41.3971 | 0.225459 |
| 2025-06 | 0 | 20.8113 | 40.833 | 0.789562 |
| 2025-07 | 0 | 20.8113 | 41.3971 | 0.225459 |

Note:Rate of unsubmitted and inappropriately submitted samples（%）= (Number of cases with specimens inappropriately submitted + Number of cases with unsubmitted)/(The total number of HAI cases)

During the baseline period (January 2022 – November 2023), the non‑conformance rate（Rate of unsubmitted and inappropriately submitted samples） fluctuated considerably, ranging from 8.16% to 60.26%, with a mean of 30.53%. Notably, a distinct peak (60.26%) was observed in December 2022, coinciding with the major policy shift in China‘s COVID‑19 response, which likely disrupted routine clinical workflows. Several baseline points exceeded the UCL, indicating a statistically unstable process.

During the intervention‑adaptation phase (December 2023 – February 2024), the non‑conformance rate exhibited a progressive downward trend (27.42% → 23.08%), reflecting the learning curve and workflow integration as the MISCP‑S model was gradually implemented.

Following full deployment of the MISCP‑S intervention (March 2024 – July 2025), the non‑conformance rate dropped sharply and consistently, with 15 out of 17 months achieving 0% non‑conformance and all 17 months remaining below the overall CL. This pattern met the SPC rule of “≥9 consecutive points on the same side of the CL” (in this case, the lower side), indicating a sustained special‑cause shift in the process attributable to the intervention. The corresponding specimen submission rate improved from 69.47% in the baseline period to 98.38% in the stable post‑intervention period (consistent with the 98.38% reported in Table 4, with minor differences due to rounding).
